# Bildgebung in der Kindertraumatologie/Kinderorthopädie

**DOI:** 10.1007/s00064-023-00839-1

**Published:** 2024-02-06

**Authors:** Theddy Slongo, Enno Stranzinger

**Affiliations:** 1 Kinderorthopädie / Kindertraumatologie, Medizinbereich Kinder und Jugendliche, Universitätsklinik für Kinderchirurgie, Juli-von-Jenner-Haus, Freiburgstr. 15, 3010 Bern, Schweiz; 2 Universitätsinstitut für Diagnostische, Interventionelle und Pädiatrische Radiologie (DIPR), Inselspital, Universitätsspital Bern, Anna-Seiler-Haus, Freiburgstr. 20, 3010 Bern, Schweiz

**Keywords:** Kinderradiologie, Strahlenbelastung, Röntgenbild, Computertomographie, Magnetresonanztomographie, Pediatric radiology, Radiation exposure, X‑ray, Computed tomography, Magnetic resonance imaging

## Abstract

Nach wie vor ist das konventionelle bzw. das digitale Röntgenbild die Grundlage der bildgebenden Diagnostik des Skelettsystems im Kindesalter. Es gilt als „Goldstandard“ in der Diagnostik, bei der Durchführung von Therapien sowie für Verlaufskontrollen. Ergänzend dazu sollen und können Verfahren wie Ultraschall, Computertomographie (CT), Magnetresonanztomographie (MRT), aber auch nuklearmedizinische Techniken eingesetzt werden. Es ist von Vorteil, dass in der Röntgendiagnostik von Kindern geschulte Radiologiefachpersonen eingesetzt werden. Im Falle von nicht spezifisch eingerichteten Kinderradiologien sollen zumindest die von Fachgesellschaften („as low as reasonable achievable“ [ALARA]) und Strahlenschutzkommissionen empfohlenen Richtlinien eingehalten werden. Es wird dargestellt, wie modernste Hilfsmittel wie Dosisüberwachungssysteme und Software-gesteuerte Bildbearbeitung und auch Nachbearbeitung sowie die verschiedenen Modalitäten optimal eingesetzt werden können, um mit möglichst geringem Aufwand und möglichst geringer Belastung für das Kind ein optimales Resultat, sprich Diagnostik, zu erreichen.

## Abstract (en)

Conventional or digital radiography is still the basis of imaging diagnostics of the skeletal system in pediatric patients. It is considered the gold standard for diagnosis, treatment selection, and follow-up. In addition, procedures such as ultrasound, computed tomography (CT), magnetic resonance imaging (MRI), and also nuclear medicine techniques can and should be used. It is advantageous to use trained radiology technicians who are familiar with the handling of children in X‑ray diagnostics. If there is no dedicated pediatric radiology department, it is recommended to follow the guidelines from radiology societies (as low as reasonably achievable [ALARA]) and radiation protection commissions. The present article describes how state-of-the-art tools such as dose monitoring systems and software-controlled image processing and also postprocessing can be used. The article provides information on how the various modalities can be optimally used in order to achieve the best result, i.e., diagnosis, with the least possible effort and burden for the child.

## Lernziele

Nach Lektüre dieses Beitrags …

- sind Sie in der Lage, die Vorteile der konventionellen Radiologie in der Akutsituation zu beschreiben,
- können Sie die Einstelltechnik bei Kindern richtig beurteilen,
- kennen Sie die bedeutendsten Indikationen der Sonographie,
- verstehen Sie die Vor- respektive Nachteile von Magnetresonanztomographie (MRT) und Computertomographie (CT),
- ist es Ihnen möglich, die Strahlenbelastung von Röntgenbildern richtig zu beurteilen,
- sind Ihnen die aktuellsten Strahlenschutzempfehlungen zum Gonadenschutz bekannt.

## Einleitung

Das konventionelle bzw. digitale **Röntgenbild**

Röntgenbild

des pädiatrischen Skelettsystems spielt nach wie vor die entscheidende Rolle für Diagnostik, Therapie und Verlaufskontrollen von Skeletttraumen und Folgen von orthopädischen Erkrankungen. Es ist die Grundlage der **bildgebenden Diagnostik**

bildgebenden Diagnostik,

die durch Verfahren wie Ultraschall, Computertomographie (CT), Magnetresonanztomographie (MRT) oder nuklearmedizinische Techniken ergänzt werden kann. Röntgenbilder sind weltweit gut verfügbar, können schnell durchgeführt werden und sind kosteneffizient.

Seit über 100 Jahren haben Röntgenbilder die Diagnostik und Therapie von skelettären Erkrankungen entscheidend geprägt und verbessert. Die Röntgentechnologie hat sich stetig weiterentwickelt, wobei die Bildqualität permanent verbessert werden konnte unter gleichzeitiger erheblicher Reduktion der **Strahlenbelastung**

Strahlenbelastung.

Ausgebildete **Fachpersonen für Radiologie**

Fachpersonen für Radiologie

(RFP), die im Umgang mit Kindern und Eltern geübt sind, erzielen eine gute Bildqualität bei geringstmöglicher Dosis, indem sie in der **Einstelltechnik**

Einstelltechnik

bei Kindern geschult sind und jeweils die individuellen Bedürfnisse des Kindes betreffend Lagerung und Einstellparameter berücksichtigen. **Fachärzte für Kinderradiologie**

Fachärzte für Kinderradiologie

optimieren stetig zusammen mit den Applikationsspezialisten und Physikern die neuesten technischen Möglichkeiten der diagnostischen Infrastruktur. **Dosisüberwachungssysteme**

Dosisüberwachungssysteme

unterstützen dabei die Optimierung und Einhaltung von Dosisreferenzwerten. Gut abgestimmte Abläufe zwischen der Abteilung für Bildgebung und kinderorthopädischer und/oder kinderchirurgischer Klinik ermöglichen eine qualitativ hochwertige, schnell verfügbare und gute bildgebende Diagnostik. Die **Bilddiagnostik**

Bilddiagnostik

ist neben der Anamnese und der klinischen Untersuchung die wichtigste Information für eine korrekte Diagnose und erfolgreiche Therapie von Frakturen und Luxationen sowie orthopädischen Erkrankungen bei Kindern. Regelmäßige radiologische Bildbesprechungen oder Rapporte erlauben eine fachliche und technische Qualitätskontrolle, spielen eine Rolle in der Weiterbildung und sind für den Patienten von großem Nutzen. Der enge klinische Austausch ist nötig für die Ausbildung von Radiologen und Orthopäden und verbessert das gegenseitige Verständnis orthopädischer Erkrankungen bei Kindern.

## Röntgenbild

Der Vorteil des Röntgenbildes ist seine gute und rasche **Verfügbarkeit**

Verfügbarkeit,

die geringe Strahlenbelastung (ein Röntgenbild der Extremitäten entspricht ca. 2–3 h natürliche Strahlenexposition), die schnelle Bildakquisition bei Kindern ohne Bewegungsartefakte und die hohe diagnostische Aussagekraft bei traumatischen Verletzungen und orthopädischen Erkrankungen. Vor allem bei Repositionen und Fixationen besticht das Röntgenbild oder Durchleuchtungsbild durch seine gute Reproduzierbarkeit und Verständlichkeit bei den meisten **Frakturen**

**Figure 1:**
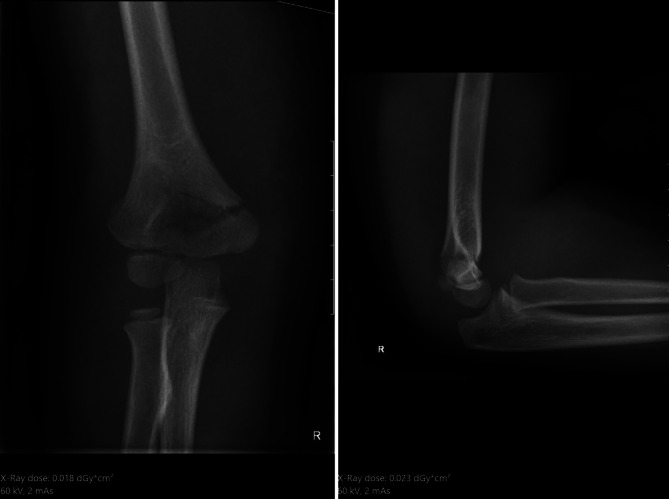

Frakturen

(Abb. 1). Das Röntgenbild kann im Gegensatz zum Ultraschall bei Frakturen **berührungsfrei**

berührungsfrei

und rasch durchgeführt werden. Zudem ist das Röntgen in einem 24-h-Klinikbetrieb wirtschaftlicher und schneller verfügbar im Vergleich zum Ultraschall, der zumeist von erfahrenen Ärzten durchgeführt werden muss.

Das Röntgenbild ist ein **Summationsbild**

Summationsbild,

wobei alle Strukturen zwischen Röntgenröhre und Detektor auf einer Ebene summiert und dargestellt werden. Zur genauen Beurteilbarkeit werden daher 2 Ebenen eines Gelenks oder einer Extremität dargestellt. Dabei können manche Frakturen bei Kindern trotz Darstellung in 2 Ebenen maskiert werden. Bei Kindern mit Trampolin‑, Torwart‑, Stress- bzw. Toddler-Frakturen sowie bei Kindsmisshandlung können manche „okkulte“ Frakturen erst im Verlauf von 7 bis 10 Tagen anhand der Kallusbildung oder Sklerose erkennbar werden.

**Figure 2:**
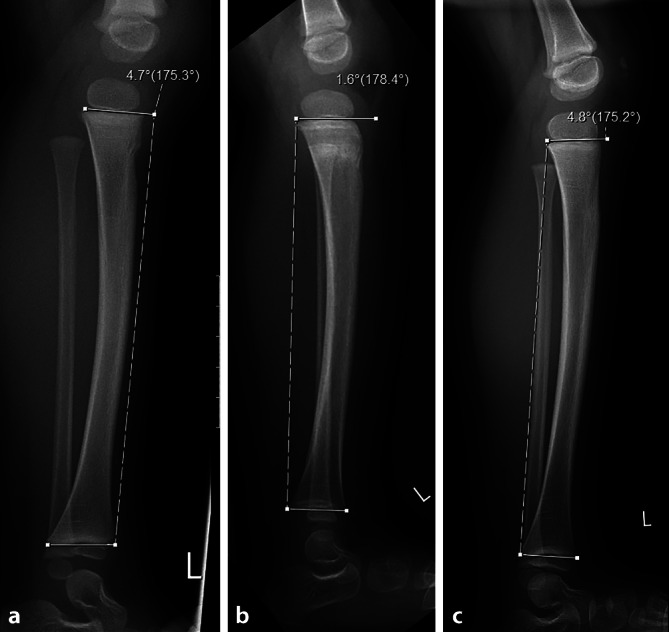

Bei Kindern können **indirekte Frakturzeichen**

indirekte Frakturzeichen

wie das Fat-Pad-Zeichen im Ellbogengelenk, das Pronator-Quadratus-Zeichen des distalen Radius oder Weichteilödeme auf eine Fraktur hinweisen. Kenntnis der normalen Tibia-Tiltwinkel, der Rogers-Hilfslinie, der Kleins-Linie oder des Radius-Wachstumsfugen-Winkels können Hinweise für Frakturen, Epiphysiolysen, Stress- oder Überlastungsfrakturen geben (Abb. 2; [1]).

### Einstelltechnik

**Figure 3:**
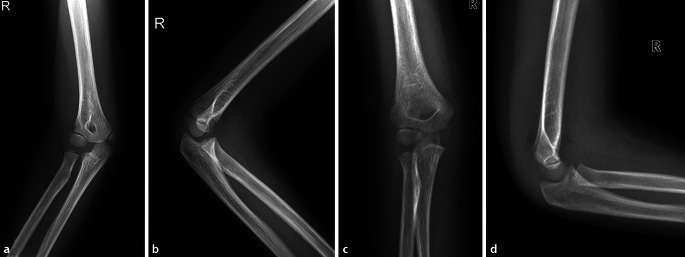

Die **korrekte Lagerung**

korrekte Lagerung

ist bei Kleinkindern eine besondere Herausforderung, daher sind die Instruktion der Eltern und des Kindes über eine korrekte Einstelltechnik, aber auch die Patientenvorbereitung und Fixation wichtig. Überlagerungen auf der zu untersuchenden Region durch den nicht mehr empfohlenen Gonadenschutz, Fixationsmaterialien, Haarspangen etc. sind auf dem Röntgenbild zu vermeiden. Sie können zu falschen Beurteilungen und/oder unnötigen Wiederholungen von Röntgenaufnahmen führen. Bei der Positionierung von Gelenken ist v. a. bei Kindern darauf zu achten, dass keine schrägen Aufnahmen gemacht werden, sondern streng 90° zueinanderstehende a.-p.- und seitliche Aufnahmen (Abb. 3). Leider werden schlecht positionierte Aufnahmen nur zu oft mit dem Vermerk „erschwerte Aufnahmebedingungen“ versehen und als solche akzeptiert. Es ist evident, dass das Kind Schmerzen/Angst hat und somit wenig kooperativ ist. Um dennoch ein optimales Bild zur Diagnosestellung bei nicht optimaler Einstelltechnik zu erhalten, ist es sinnvoll, dass der behandelnde Arzt sich mit der verantwortlichen Radiologiefachperson (RFP) abspricht, wie die Schmerzen minimiert werden können, z. B. durch Kippen der Röntgenröhre.

**Figure 4:**
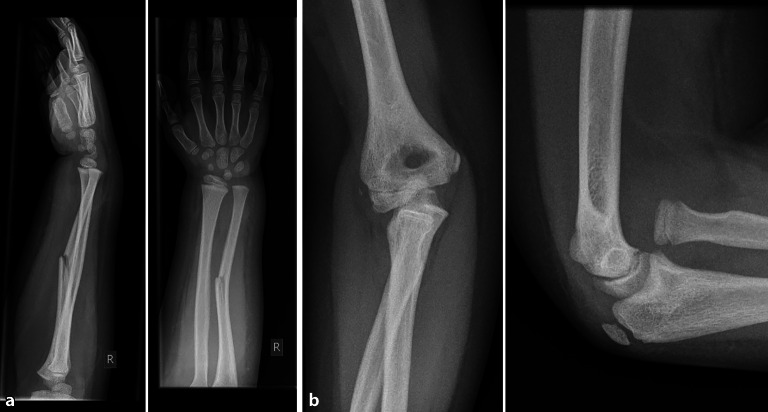

Bei Kleinkindern unter 6 Jahren, die die Lokalisation einer diaphysären Verletzung nicht sicher angeben können, ist darauf zu achten, dass die **angrenzenden Gelenke**

angrenzenden Gelenke

vollständig miterfasst werden. Somit können Rotationsfehlstellungen z. B. der Tibia oder Luxationen des Radius besser beurteilt werden. Eine ungenügende Darstellung des Ellenbogengelenks kann bei verpassten Radiusluxationen und Monteggia-Frakturen zu erheblichen Spätfolgen führen (Abb. 4).

Im Kompendium der Röntgeneinstelltechnik und Röntgenanatomie von H.P. Nowak und anderen Standardwerken der Einstelltechnik werden die genauen Einstellungen und die **Qualitätskriterien**

Qualitätskriterien

eines guten Röntgenbildes bei Kindern beschrieben [2]. Dabei wird einerseits die Lagerung der Extremitäten beschrieben, die Einstellung des Zentralstrahls sowie Kriterien für die gute Aufnahme werden definiert. Häufige Fehler sind die fehlende Einsehbarkeit in die Gelenke im Seitenbild (z. B. Ellenbogen) und die ungenügende Symmetrie der Kondylen im a.-p.-Bild (Knie, Ellenbogen). Es sei hier auf die Möglichkeit hingewiesen, dass man auch die Röntgenröhre schwenken kann, wenn das Kind eine korrekte Lagerung wegen Schmerzen oder Fehlstellung nicht einnehmen kann. Bei solchen „Varianten“ der Einstelltechnik ist es von Vorteil, wenn der behandelnde Kinderorthopäde unterstützend beraten kann.

Bei den Röntgengeräten ist zudem auf eine **optimierte Kollimation**

optimierte Kollimation

(Einblendung) zu achten, auf **altersadaptierte Einstellparameter**

altersadaptierte Einstellparameter

(Tab. 2 und 3) und eine für Kinder angepasste Nachbearbeitung (Postprocessing).

Beim **Postprocessing**

**Figure 5:**
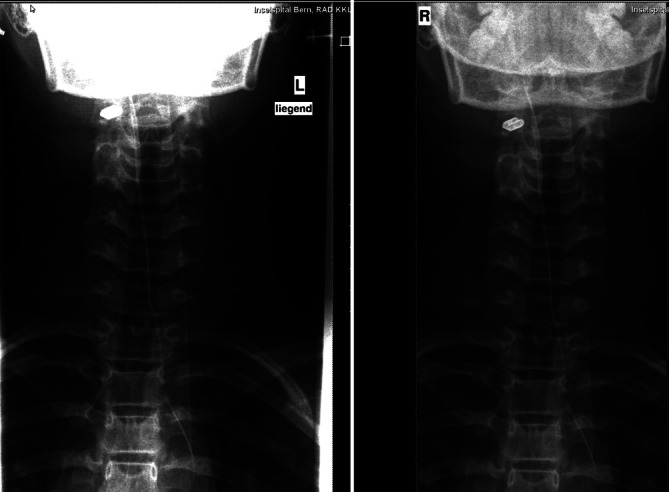

Postprocessing

bei digitalen Röntgenanlagen gilt es zu beachten, dass die Einstellungen bei Kindern optimiert werden, um Aufhärtungen z. B. des Gesichtsschädels zu minimieren, um z. B. den Dens axis beurteilen zu können (Abb. 5) bzw. die Weichteile bei Gelenken gut einsehen zu können (Abb. 1). Dies ist hilfreich zur Darstellung von Gelenkergüssen oder Weichteilödemen.

**Figure 6:**
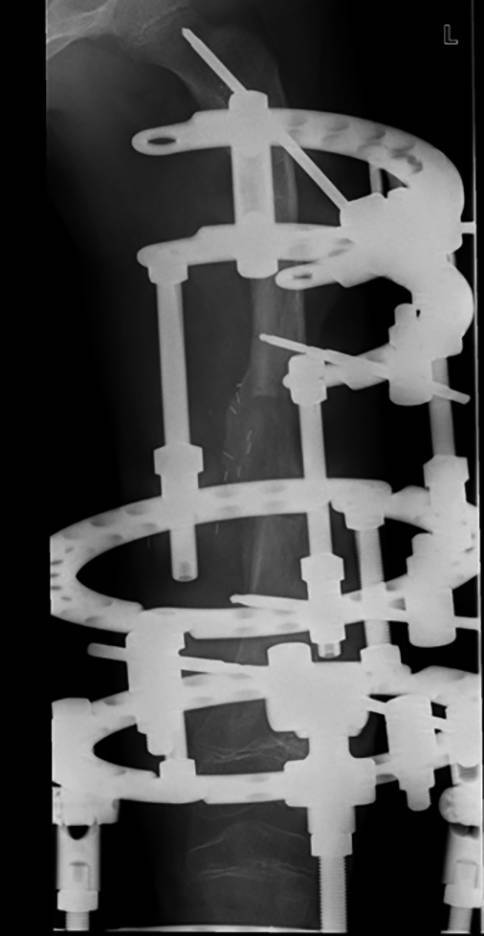

Im Weiteren lässt das Postprocessing auch Hardware-Artefakte respektive Überlagerungen, wie wir sie besonders bei Verwendung von externen Fixateuren sehen, so verändern, dass wir das Wesentliche, nämlich den Knochen oder die Knochenheilung korrekt beurteilen können. Dies ist in Abb. 6 dargestellt.

## Sonographie

Die Sonographie von Frakturen und Gelenkpathologien („point-of-care ultrasound“ [POCUS]) hat in vielen pädiatrischen und chirurgischen Praxen und Notfallstationen Einzug gehalten und mag bei unkomplizierten Verletzungen oder zum Ausschluss von Frakturen genügen. Der Ultraschall ist zusätzlich zum Röntgenbild hilfreich zur Bestätigung von Gelenkergüssen, knorpeligen, ligamentären oder muskulären Verletzungen. Die Ultraschallgeräte werden immer günstiger, kleiner und portabler, sodass sich ein **Bedside-Einsatz**

**Figure 7:**
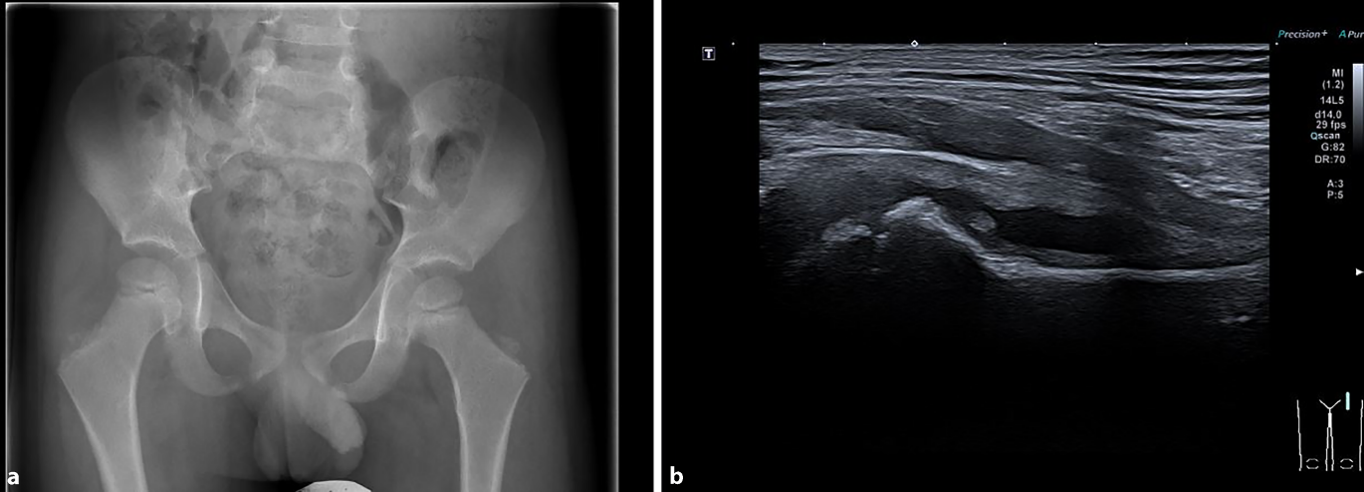

Bedside-Einsatz

anbietet. Dennoch bleibt der Ultraschall **untersucherabhängig**

untersucherabhängig

und bedarf guter anatomischer Kenntnisse des muskuloskeletalen Systems bei wachsenden pädiatrischen Knochen und deren zahlreichen Varianten (Abb. 7). Einen Vorteil der Sonographie stellt die Möglichkeit der **dynamischen Untersuchung**

dynamischen Untersuchung

dar. Diese ermöglicht, z. B. Sehnenrupturen, Muskelaktivitäten und Gelenkbewegungen darzustellen. Zusätzlich kann der Ultraschall die Durchblutung von Weichteilprozessen oder vaskulären Fehlbildungen im Doppler darstellen, die Steifigkeiten von Geweben mit der Elastographie bestimmen und mit i.v.-Kontrastmittelgabe die Dynamik von vaskularisierten Läsionen beschreiben.

Der Ultraschall hat sich für **Vorsorgeuntersuchungen**

Vorsorgeuntersuchungen

bei Kindern mit Risikofaktoren, insbesondere bei Verdacht auf Hüftdysplasie, bewährt. Der Ultraschall vermag auch zur Beurteilung von Erb-Lähmungen die Stellung des Humeruskopfes beschreiben und kann für Patella- und Trochleadysplasien im Neugeborenenalter verwendet werden. Bei Neugeborenen mit Tortikollis kann der Ultraschall einfach eine Fibromatosis colli darstellen. Auch Segmentationsstörungen z. B. im Rahmen der Abklärung eines „tethered cord“ sind im fetalen Ultraschall oder Neugeborenenalter gut erkennbar.

Der Ultraschall hat Vorteile bei Kleinkindern mit noch knorpliger Anlage der Epiphysen [3]. In vielen Fällen ergänzt der Ultraschall das Röntgenbild und kann in geübten Händen zu einer höheren diagnostischen Sicherheit führen.

## Computertomographie

**Figure 8:**
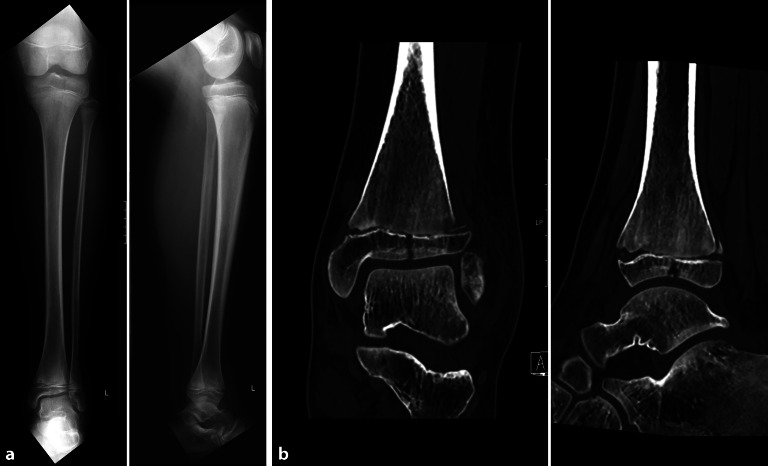

Die Computertomographie (CT) spielt bei **komplexen Frakturen**

komplexen Frakturen

eine wichtige Rolle zur Operationsplanung und ermöglicht eine 3‑dimensionale Volumen-Rendering-Darstellung sowie eine tomographische Rekonstruktion in allen Ebenen (Abb. 8). Die CT wird auch zunehmend zur Darstellung von Frakturen der Hand- und Fußwurzelknochen als auch des Gesichtsschädels eingesetzt und hat tomographische Röntgenaufnahmen oder Spezialaufnahmen (z. B. Skaphoid-Quartett) weitestgehend abgelöst. Dabei gibt es kleine **Extremitäten-CT-Scanner**

Extremitäten-CT-Scanner,

die auch stehende Aufnahmen des oberen Sprunggelenkes (OSG) erlauben und nur eine begrenzte respektive limitierte Exposition der Extremitäten verursachen.

In der CT können zudem mit **Kontrastmittelgabe**

Kontrastmittelgabe

angiographische Gefäßrekonstruktionen dargestellt werden. Moderne Dual-Energy- und Photon-Counting-CT ermöglichen spezifische **Gewebecharakterisierungen**

Gewebecharakterisierungen

wie Gichtdarstellungen, virtuelle Nativsequenzen, aber auch Knochenödemdarstellungen. Die **Strahlenbelastung**

Strahlenbelastung

in der CT hat mit den aktuellen CT-Generationen durch Einsatz von iterativen Rekonstruktionsmöglichkeiten, automatischer Dosismodulation, modernen Detektoren, Filtern und neuerdings Photon-Counting-Detektoren signifikant abgenommen bei besserer Bildqualität.

Die **CT-Bilddateninformationen**

**Figure 9:**
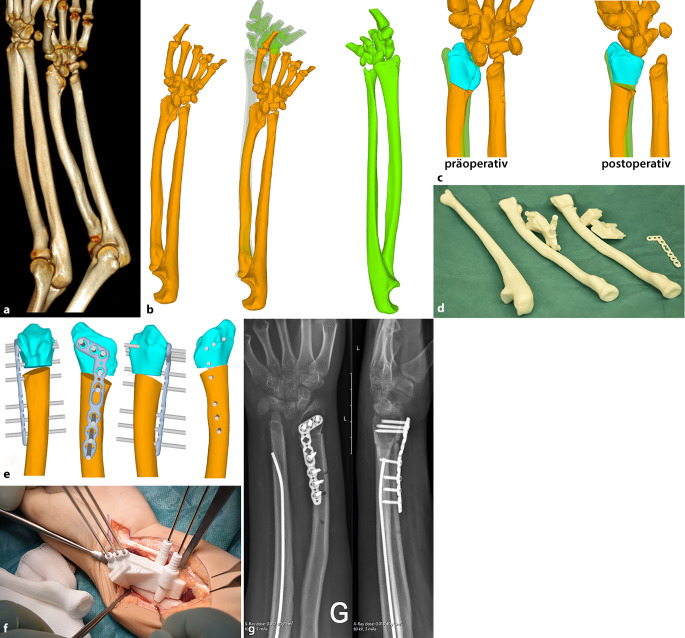

CT-Bilddateninformationen

des Knochens sind gut geeignet zur Segmentierung und Nachbearbeitung und können für 3‑D-Drucke verwendet werden („standard triangulation language file“ [stl]). Dies kann eine Operationsplanung, eine Operationssimulation durch Schablonen, eine augmentierte Realität durch Bildfusionen oder eine Behandlung mit individualisierten Prothesen ermöglichen (Abb. 9). Zur genauen automatischen Segmentierung ist jedoch für **3‑D-Drucke**

3‑D-Drucke

häufig eine höhere Dosis zur Bildakquisition nötig als für rein diagnostische, pädiatrische CT-Untersuchungen des Skelettsystems. Das heißt, die höhere Strahlenbelastung muss mit einem therapeutischen Mehrwert für den Patienten einhergehen, der durch den 3‑D-Druck erzielt wird. Eine CT-Untersuchung mit Anforderung für einen 3‑D-Druck ohne diagnostischen oder therapeutischen Nutzen ist zu vermeiden. MR(Magnetresonanz)-Daten mit dem höheren Weichteilkontrast sind schwieriger nutzbar für 3‑D-Drucke des Skelettsystems und bedürfen meist einer sehr zeitintensiven, manuellen Segmentierung.

## Magnetresonanztomographie

**Figure 10:**
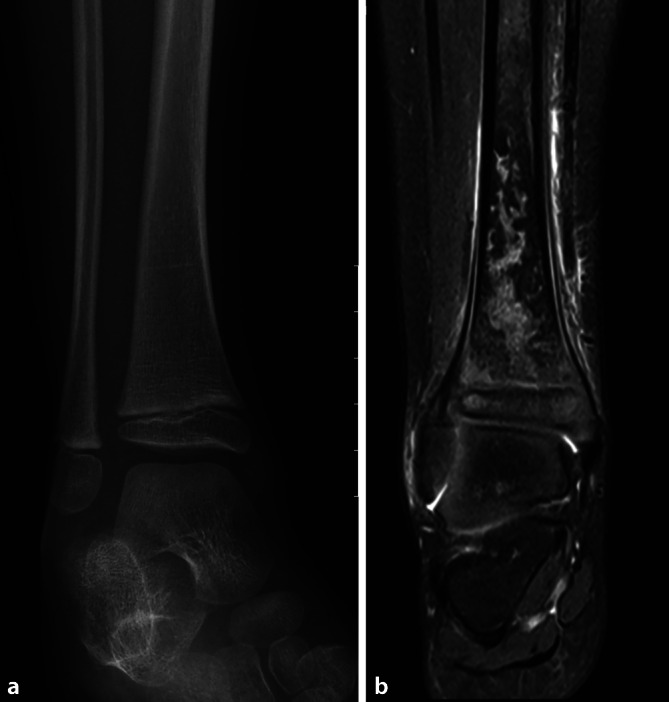

Die Stärken der Magnetresonanztomographie (MRT) sind der **hervorragende Weichteilkontrast**

hervorragende Weichteilkontrast

(Knorpel, Menisken, Labren, Bänder und Sehnen) und die gute Beurteilbarkeit des Knochenmarks bei Kindern (z. B. Leukämie, Infarkte, Tumoren, Entzündungen) (Abb. 10). Fragestellungen zur Vaskularisation des Knochens z. B. bei Epiphysiolysen des Hüftkopfes oder Skaphoidfrakturen können mit der **Kontrastmittel-unterstützten MRT**

Kontrastmittel-unterstützten MRT

beantwortet werden. Time-to-peak-Kurven bei Knochentumoren erlauben zusammen mit diffusionsgewichteten Sequenzen eine genauere Differenzierung von Tumoren. Moderne Knorpelsequenzen ermöglichen eine 3‑dimensionale morphologische und biochemische Analyse des Gelenkknorpels.

In der akuten Phase eines Skeletttraumas hingegen hat die MRT häufig keine klinischen Konsequenzen, da das akute Knochen- und Weichteilödem das wahre Ausmaß der Verletzung überschätzt. Zudem brauchen Kleinkinder unter 6 Jahren häufig eine Sedation für die Untersuchung. Neuere KI(**künstliche Intelligenz**

künstliche Intelligenz

)-gestützte Bildrekonstruktionstechnologie erlaubt einen MR-Scan des Kniegelenkes in 2 min und kann künftig die Untersuchungszeiten verkürzen und die Notwendigkeit von Sedationen bei Kindern deutlich vermindern.

**Ganzkörper-MRTs**

Ganzkörper-MRTs

haben in der Kinderradiologie manche szintigraphische Methode abgelöst wie z. B. bei der Suche nach Histiozystoseläsionen, Darstellung von Lymphomen, Metastasen, entzündlichen multifokalen Läsionen (chronisch rekurrierende multifokale Osteomyelitis [CRMO]), rheumatischen Erkrankungen. Sie können zur Suche von Skelettverletzungen bei Kindesmisshandlung ergänzend hilfreich sein.

**Figure 11:**
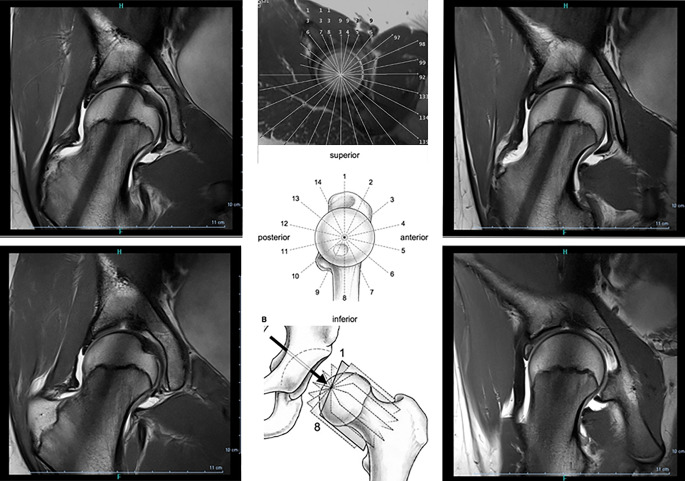

In der MRT können **beliebige Ebenen**

beliebige Ebenen

geplant werden, so sind auch radiäre Schichten durch Schenkelhals und Hüftkopf möglich (Abb. 11), wobei Fragen nach Impingement und Offset des Schenkelhalses beantwortet werden können. Nur bei sehr dünnen MR-Sequenzen sind analog zur CT auch Rekonstruktionen möglich. Eine Limitation bleibt die Bildgebung des Skeletts bei metallischen Implantaten, die in der Regel erhebliche **Artefakte**

Artefakte

verursachen. Hier können sog. **Wrap-Sequenzen**

Wrap-Sequenzen

zur Reduzierung solcher Metallartefakte benutzt werden.

## Knochenszintigraphie

Eine Knochenszintigraphie ist ein nuklearmedizinischer Test, der mithilfe von **radioaktiv markierten Tracern**

radioaktiv markierten Tracern

verschiedene Arten von Knochenerkrankungen diagnostiziert. Der Tracer wird von Knochenzellen und Geweben, die sich schnell umbauen, stärker absorbiert. So kann ein Knochenscan dazu dienen, die Ursache für Skelettschmerzen, eine Knocheninfektion, einen Tumor, Metastasen oder eine Knochenverletzung zu finden, die auf einem normalen Röntgenbild nicht zu erkennen ist.

## Positronenemissionstomographie

Bei der Positronenemissionstomographie (PET) werden kleine Mengen radioaktiver Materialien, sog. Radiotracer oder Radiopharmaka, eine spezielle Kamera und ein Computer verwendet, um Organ- und Gewebefunktionen zu beurteilen. Durch die Identifizierung von Veränderungen auf zellulärer Ebene kann die PET den frühen Beginn einer Krankheit erkennen. Dabei wird in der Regel ein **radioaktiv markiertes Glukoseanalogon**

radioaktiv markiertes Glukoseanalogon

(FDG[F-18-Fluorodesoxyglukose]-PET) verwendet und ist zum Staging verschiedener Tumoren geeignet. Die **PET-CT**

PET-CT

ordnet die pathologischen Stoffwechselveränderungen anatomischen Strukturen in der CT zu.

## Strahlenschutz

Kinder unterscheiden sich von erwachsenen Patienten durch ihre geringe Größe und ihre erhöhte Empfindlichkeit gegenüber ionisierender Strahlung. Zudem haben Kinder eine längere Lebenserwartung als Erwachsene und können einen theoretischen **Strahlenschaden**

Strahlenschaden

erleben. Daher haben Kinder ein besonderes auf Anrecht eine individuelle Anpassung der physikalischen Parameter (kV [Kilovolt], mAs [Milliamperesekunde], Filter) der Röntgenanlage bei sehr variierenden Gewichtsgruppen, um das pädiatrische Skelettsystem mit einer optimalen Bildqualität bei gleichzeitig tiefstmöglicher Strahlenbelastung darzustellen. Dabei sind die Grundlagen des Strahlenschutzes, wie sie bei der CT häufig erwähnt werden, auch bei allen anderen Röntgenuntersuchungen, Durchleuchtungsuntersuchungen und intraoperativen Bildverstärkeranlagen anzuwenden (Tab. 1).

**Table 1:** 

1. Indikation prüfen
2. Keine unnötigen Wiederholungen oder Vergleichsbilder der Gegenseite
3. Enges Einblenden auf die zu untersuchende Region; die Blenden müssen auf dem Bild sichtbar sein
4. Prüfe Alternativen wie Ultraschall (US) oder Magnetresonanztomographie (MRT)
5. Mögliche Schwangerschaften ausschließen
6. Es muss nicht das perfekte Röntgenbild mit höchster Dosis sein, sondern ein diagnostisches Bild
7. Nutze patientenadaptierte Parameter (kV, mAs) und entsprechende Filter (Cu, Al)
8. Zentrierte das Gelenk in der Mitte des Bildes
9. Dokumentiere die Röntgendosis im Bericht bei Durchleuchtungen und Röntgenuntersuchungen der Wirbelsäule und vergleiche sie regelmäßig mit Referenzwerten (s. Tab. 2)

*kV* Kilovolt, *mAs* Milliamperesekunde, *Cu* Kupfer, *Al* Aluminium

**Figure 12:**
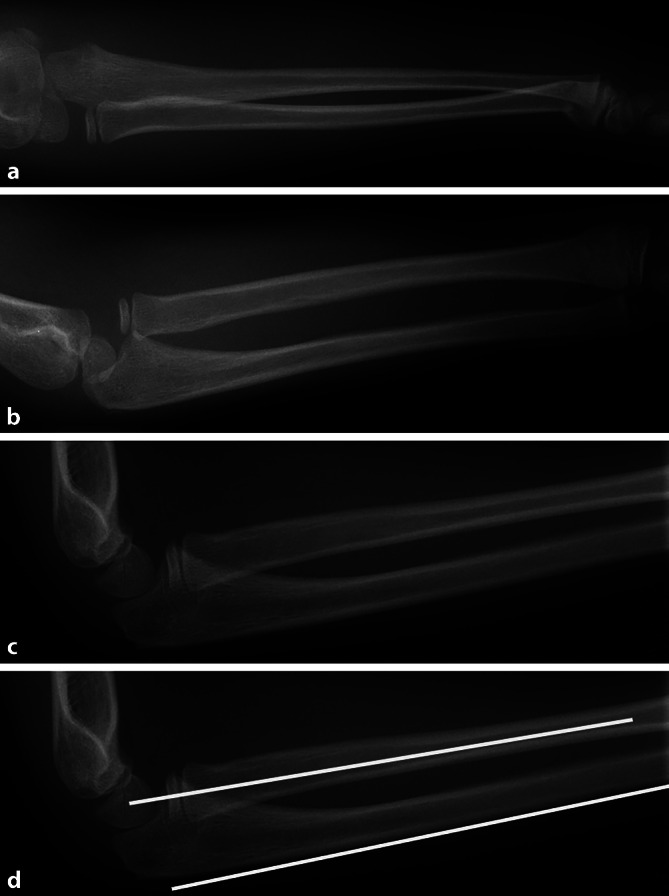

Der beste Strahlenschutz besteht darin, die Indikation und die Notwendigkeit einer Röntgenuntersuchung streng zu prüfen und auch Alternativen in Erwägung zu ziehen, die mit gleicher diagnostischer Sicherheit, Verfügbarkeit und Wirtschaftlichkeit die Fragestellung beantworten können. Jede Röntgenuntersuchung soll eine klinische Konsequenz nach sich ziehen, eine Effektivität nachweisen, die einen Nutzen für den Patienten bedeutet und die Strahlenbelastung rechtfertigt. Zu vermeiden sind Vergleichsaufnahmen der Gegenseite (Abb. 12) oder zu frühe oder zu engmaschige oder sogar unnötige Verlaufskontrollen bei stabilen Frakturen oder sehr engmaschige Skolioseverlaufskontrollen [4].

Bei der Durchführung der Untersuchung müssen die **Qualitätsrichtlinien**

Qualitätsrichtlinien

bei Kindern gemäß nationaler oder internationaler Richtlinien, aktueller Referenzwerten und Verordnungen eingehalten werden.

Es besteht weiterhin eine sehr heterogene Infrastruktur der Röntgengeräte im Gesundheitswesen weltweit von Film-Folien-Systemen über konventionelle Röntgensysteme bis zu modernen digitalen Röntgenanlagen. Computer Radiographie(CR)-Anlagen ermöglichen eine bis zu 50 % niedrigere Dosis im Vergleich zu Film-Folien-Systemen, die kaum mehr in Betrieb sind. Die modernen **digitalen Röntgenanlagen**

digitalen Röntgenanlagen

mit Flat-Panel-Detektoren erreichen im Vergleich zu Computer Radiographie-Anlagen (CR-Anlagen) nochmals eine bis zu 50 % niedrigere Dosis und erlauben ein besseres Postprocessing. Entscheidend in der pädiatrischen Radiologie ist die Verwendung eines **Kupferfilters**

Kupferfilters

und **Aluminiumfilters**

Aluminiumfilters

zur Dosisreduktion [5]. Zudem wird empfohlen, Voreinstellungen auf den Geräten zu hinterlegen, die nach Gewichtsklassen oder Altersgruppen festgelegt werden können (Tab. 2 und 3). Eine **individualisierte Einstellung**

individualisierte Einstellung

der kV- und mAs-Parameter gemäß Gewicht und Größe kann die Dosis entscheidend reduzieren.

**Table 2:** 

Durchschnitt (*n* = 100) DFP in cGY*cm^2^	Neonaten < 5 kg + < 1 Monat	Kleinkind 5–15 kg + 1 Monat bis 4 Jahre	Mittlere Kindheit 15–30 kg + 5 bis 10 Jahre	Frühe Adoleszenz 30–50 kg + 10 bis 15 Jahre	Späte Adoleszenz > 50 kg + 14 bis 16 Jahre
Schulter a.-p. Schulter lat.	–	0,32	0,68	1,08	1,67
		0,48	1,56	2,28	3,44
Oberarm a.-p. Oberarm lat.	0,18	0,38	0,66	1,33	2,23
	0,08	0,26	1,05	2,15	4,83
Ellenbogen a.-p. Ellenbogen lat.	–	0,15	0,20	0,32	0,36
		0,16	0,22	0,26	0,36
Vorderarm a.-p. Vorderarm lat.	–	0,19	0,33	0,53	0,69
		0,20	0,31	0,51	0,65
Handgelenk a.-p. Handgelenk lat.	–	0,11	0,18	0,26	0,32
		0,11	0,15	0,21	0,26
Hand a.-p. Hand schräg	0,29	0,23	0,36	0,54	–
		0,19	0,36	0,48	
Femur a.-p. Femur lat.	0,39	1,07	2,85	4,95	7,52
	0,19	0,92	2,87	4,79	7,32
Knie a.-p. Knie lat.	0,12	0,24	0,48	0,77	1,41
	0,09	0,27	0,58	0,89	1,53
Unterschenkel a.-p. Unterschenkel lat.	0,22	0,41	0,84	1,49	2,20
	0,11	0,43	0,95	1,63	2,26
OSG a.-p. OSG lat.	–	0,16	0,31	0,48	0,72
		0,18	0,41	0,63	0,97
Fuß a.-p. Fuß schräg	0,16	0,22	0,36	0,57	0,70
	0,16	0,24	0,41	0,63	0,80

*DFP* Dosis-Flächenprodukt, *a.-p.* anterior-posterior, *lat.* lateral, *OSG* oberes Sprunggelenk

**Table 3:** 

Beschreibung	Gewichtsgruppe	Alter
Neonaten	< 5 kg	< 1 Monat
Kleinkinder	5–15 kg	1 Monat bis 4 Jahre
Mittlere Kindheit	15–30 kg	5 bis 10 Jahre
Frühe Adoleszenz	30–50 kg	10 bis 15 Jahre
Späte Adoleszenz	50–80 kg	14 bis 18 Jahre

Für nicht unmittelbar beteiligte Personen im Operationssaal oder Röntgenraum gilt: Es sollen sich nur die Personen im Raum aufhalten, die für die Operation oder Untersuchung nötig sind, es soll das **Abstands-Quadrat-Gesetz**

Abstands-Quadrat-Gesetz

eingehalten werden (möglichst weite Entfernung zur BV[Bildverstärker]-Anlage), und es können Bleischürzen und Trennwände eingesetzt werden, um die Dosis für den Untersucher zu minimieren. Die Größe des Durchleuchtungsfeldes soll auf die zu interessierende Region eingeblendet werden. Helfende Hände zur Lagerung sollen möglichst nicht im Bild aufgenommen werden.

Die neuesten Richtlinien zum Strahlenschutz empfehlen, **keinen Gonadenschutz**

keinen Gonadenschutz

mehr bei Kindern bei Abdomen- oder Beckenröntgenbildern zu verwenden. Die durch den Gonadenschutz verursachte **Streustrahlung**

Streustrahlung

bewirkt eine höhere Strahlenbelastung für strahlenempfindliche Gewebe wie Magen und Darm. Die Ovarien bei Mädchen werden wegen der variablen anatomischen Lage häufig nicht geschützt oder abgedeckt. Zudem verdeckt der Gonadenschutz manchmal wesentliche Pathologien, Frakturen oder Entzündungen des Beckenringes und des Sakrums [6, 7]. Zudem wird der Wichtungsfaktor für Gonaden nicht mehr so hoch eingestuft wie früher, sodass die neuesten europäischen und amerikanischen Empfehlungen auf einen Gonadenschutz bei Kindern gänzlich verzichten.

### Einstellparameter

**Figure 13:**
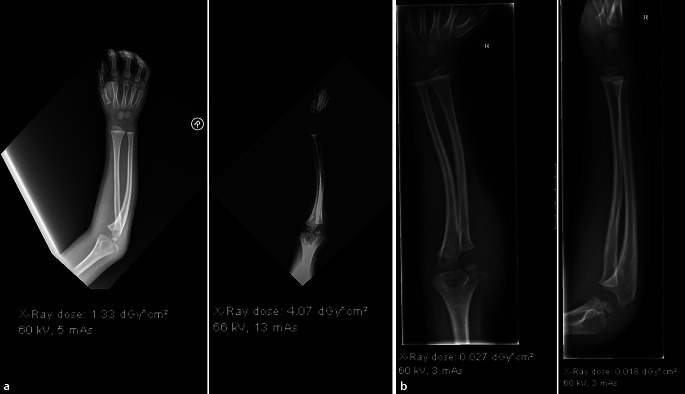

Eine ausgebildete Fachperson für Radiologie, die im Umgang mit Kindern und Eltern geübt ist, kann durch die richtige Einstelltechnik, das korrekte Einblenden auf die zu untersuchende Extremität und die Verwendung der alters- und gewichtsadaptierten Scanparameter deutlich zum Strahlenschutz und gleichzeitig zu einer besseren Bildqualität beitragen. Ein optimal durchgeführtes Röntgenbild in 2 Ebenen erhöht die diagnostische Aussagekraft und vereinfacht die Interpretation bei gleichzeitig besserer Bildqualität (Abb. 13).

Obwohl ein deterministischer Strahlenschaden durch eine einmalige Röntgenuntersuchung unwahrscheinlich ist, so kann durch häufige Verlaufskontrollen wie bei Skolioseröntgenuntersuchungen eine **stochastische Strahlenwirkung**

stochastische Strahlenwirkung

möglich sein. Die Strahlenbelastung sollte daher für jede Röntgenuntersuchung so niedrig wie möglich gehalten werden. Viele Hersteller machen sich dieses Strahlenrisiko v. a. bei Kindern zunutze, z. B. bei **Low-dose-Ganzkörperscannern**

Low-dose-Ganzkörperscannern.

**Figure 14:**
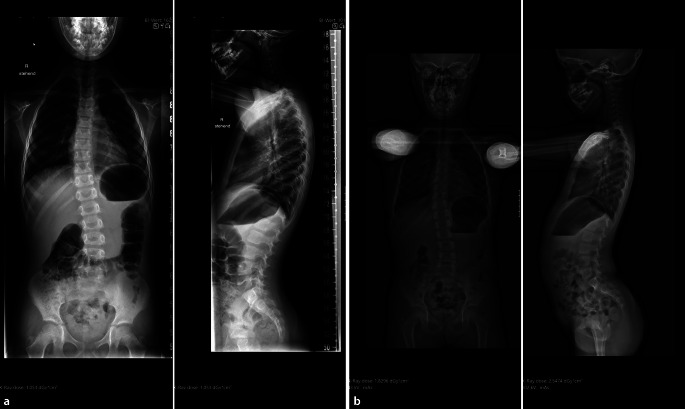

Die Strahlenbelastung dieser Geräte bei kleineren Kindern kann im Vergleich zu einem korrekt angefertigten Röntgenbild mit angepassten pädiatrischen Parametern dennoch höher liegen (Abb. 14). Somit kann das konventionelle digitale Röntgenbild weiterhin die Modalität mit der geringstmöglichen Dosis sein, wenn es für Kinder optimiert ist.

In den letzten Jahrzehnten haben viele technische Innovationen der Röntgen‑, Durchleuchtungs- und CT-Geräte zu einer erheblichen Strahlenreduktion im gesamten Bereich der Kinderradiologie geführt. Zudem konnte dank des Ultraschalls und der MRT das Strahlenrisiko bei Kindern so weit reduziert werden, dass kein Schaden durch die Bildgebung für den Patienten zu erwarten ist. Ein **Dosisüberwachungssystem**

Dosisüberwachungssystem

ist empfehlenswert, um die Röntgendosis von verschiedenen Gewichts- oder Altersgruppen jeweils optimieren zu können und die Parameter und Strahlenbelastung an neue Geräte oder Innovationen (z. B. Photon-Counting-Detektoren) anpassen zu können. Um die korrekte effektive Dosis dem Patienten zuordnen zu können, ist es notwendig, für jede Röntgenuntersuchung das Gewicht und die Größe des Patienten für das Dosisüberwachungssystem zu erfassen. Diese Werte können automatisch von der Messstation an das System übermittelt und regelmäßig für verschiedene Gewichtsgruppen kontrolliert werden. Dabei spielen **Dosisreferenzwerte**

Dosisreferenzwerte

eine immer wichtigere Rolle, die mit dem Dosisüberwachungssystem einfach kontrolliert und eingehalten werden können.

Die Röntgenbilder neuerer Geräte sollen dabei immer eine gleich gute oder bessere diagnostische Qualität bei möglichst niedriger Dosis im Vergleich zu der Vorgängerversion aufweisen. Häufig sind keine **kindgerechten Parameter**

kindgerechten Parameter

von Herstellerseite vorhanden, sodass man sich mit jedem neuen Gerät zusammen mit den Applikationsspezialisten und Physikern für die verschiedenen Alters- bzw. Gewichtsgruppen festlegen und jeweils die entsprechende Bildqualität beurteilen muss. Es empfiehlt sich, die in Tab. 3 aufgeführten Gewichts- oder Altersgruppen bei Kindern einzuteilen und für jedes Gerät festzulegen.

**Figure 15:**
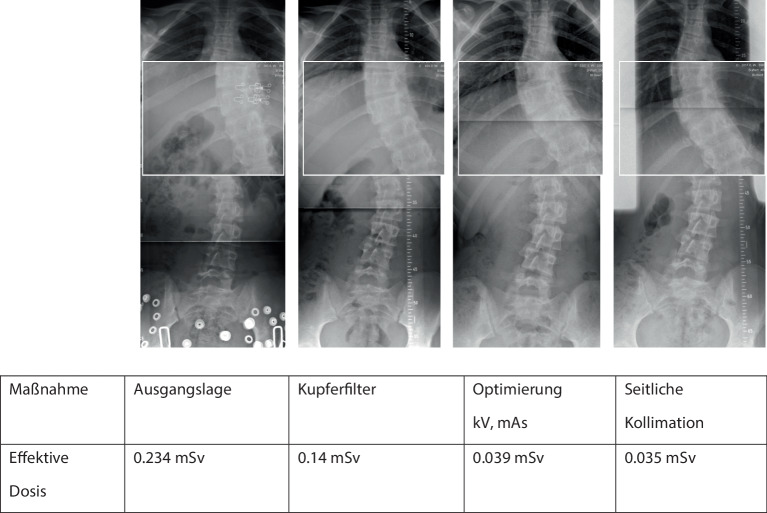

Dank des Dosisüberwachungssystems ist es in unserer Röntgenabteilung gelungen, die Strahlenbelastung von Skolioseröntgenbildern um 85 % bei gleichbleibend guter Bildqualität zu reduzieren (Abb. 15). Die Standardabweichungen der Strahlenbelastung konnten auch deutlich reduziert werden. Dazu beigetragen hat eine systematisches und stufenweises Optimieren der Röntgenuntersuchung unter Verwendung eines Kupferfilters, der Optimierung von kV- und mAs-Parametern und der Einführung einer seitlichen Kollimation der Mammae [5].

Ein bleibendes Problem ist die eingeschränkte oder fehlende Kooperation von Kleinkindern (unter 6 Jahren) während der Röntgenuntersuchung. Vor allem Kinder mit Verletzungen haben Schmerzen und Angst und sind schwierig zu überzeugen, während der Röntgenuntersuchung die korrekte Lagerung einzuhalten. Daher sind eine gute Führung, Lagerung und Fixation der Kinder und ein aktiver Einbezug der Eltern notwendig, um ein korrektes Röntgenbild zu erreichen. Ist zur Sicherstellung der Diagnose ein optimales Röntgenbild unerlässlich, sollte mit den Eltern sowie mit dem Röntgenpersonal über eine mögliche **Analgosedation**

Analgosedation

diskutiert werden. In diesem Fall muss eine entsprechende Überwachung während der Untersuchung sowie je nach regionalen Richtlinien 1–2 h über die Untersuchung hinaus gewährleistet sein.

Eine **gute Kooperation**

gute Kooperation

und **Vorbereitung**

Vorbereitung

des Kindes hingegen ermöglichen die optimale Lagerung und Einstellung des Gelenkes sowie die richtige Kollimation auf die zu untersuchende Region. Dies trägt wesentlich zur Dosisreduktion bei und zur besseren Beurteilbarkeit der Pathologie. Alle Fremdkörper, sichtbaren Lagerungshilfen, Gipse sollten möglichst aus dem Untersuchungsgebiet entfernt werden.

Eine Wiederholung der Untersuchung oder ein Seitenvergleich der gesunden Seite sollte möglichst vermieden werden (Abb. 12). Es gibt Atlanten oder Websites, um ein normales Gelenk von Kindern in verschiedenen Altersklassen zu vergleichen [2, 8].

## Betrieb von Bildverstärkeranlagen

BV(Bildverstärker)-Anlagen werden häufig in der Notfallaufnahme für Repositionen und im Operationssaal für geschlossene Repositionen und intraoperative Stellungskontrollen verwendet. Der Vorteil der **intraoperativen Durchleuchtung**

intraoperativen Durchleuchtung

besteht darin, dass der C‑Bogen immer so gekippt werden kann, dass perfekte a.-p./laterale oder falls notwendig auch gezielte Schrägaufnahmen gemacht werden können. Somit sollte es zum Standard werden, dass nach einer Reposition zur Dokumentation perfekte Bilder gemacht werden, die dann (heute meist vorhanden) ins PACS („Picture Archiving and Communication System“) eingelesen werden. Dies erübrigt dann nochmalige konventionelle Röntgenaufnahmen, häufig dann mit Gipsverband, was die Beurteilungsqualität immer vermindert.

Die dabei zu beachtenden Punkte sind in Tab. 4 aufgeführt.

**Table 4:** 

1. Kinder gut lagern und BV auf die zu untersuchende Region zentrieren
2. Keine Lagerungshilfen, Fremdkörper, Leitungen im Untersuchungsgebiet
3. Nur im Fluoro-Modus arbeiten (nicht Cine oder Exposition)
4. Kürzestmögliche Pulsationsrate, gepulste Durchleuchtung
5. Einblenden auf relevante Anatomie
6. Keine Vergrößerung
7. Nur sehr kurze Durchleuchtungssequenzen durchführen und als Film oder „last image hold“ aufnehmen
8. Bei Übertischröhren optimale hohe Lage der Röhre, Lichtvisier nutzen
9. Bei Untertischröhren möglichst nahe Position der BV-Anlage beim Objekt
10. Keine Wiederholungen des Gleichen
11. Standardisierte Abläufe/Ebenen

### Intraoperative Bildverstärkeranlagen

Auch bei intraoperativem Gebrauch von Röntgengeräten, hier v. a. der Durchleuchtungsgeräte, muss unbedingt auf entsprechende **Einblendung**

**Figure 16:**
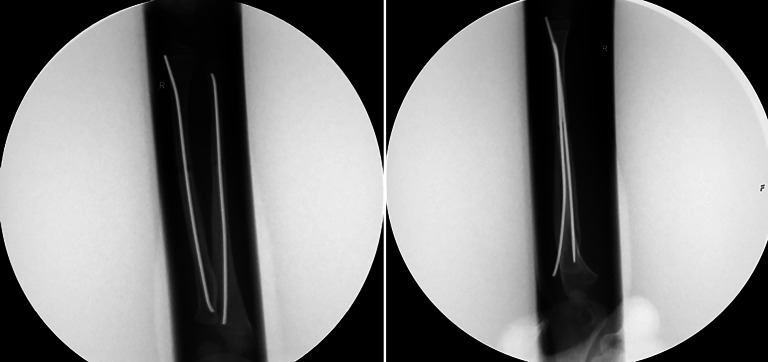

Einblendung

(Kollimation) geachtet werden (Abb. 16), zudem sollten die Hände des Operateurs ebenfalls nicht im Strahlengang liegen. Für jede Operation oder Reposition mit BV-Anlage müssen im Operationsbericht die in Tab. 5 aufgeführten Angaben vorhanden sein.

**Table 5:** 

Patienten-ID
Eingriff
Datum
Dosisflächenprodukt
Durchleuchtungszeit

## Indikationen

Häufige Indikationen in der Kinderorthopädie und -traumatologie für eine bildgebende Diagnostik sind:

- Verletzungen,
- entzündliche Veränderungen,
- Tumordiagnostik,
- angeborene Fehlbildungen und Skelettdysplasien,
- Abklärungen von Kindesmisshandlung mit standardisiertem Skelettstatus,
- Beinlängendifferenzen,
- Skoliosen
- etc.

Die klinische Fragestellung, das Alter, die Verfügbarkeit der Diagnostik und die Dringlichkeit beeinflussen die Wahl der bildgebenden Modalität [9].

Bei Kleinkindern mit **Schonhinken**

Schonhinken

nach viralem Infekt kann ein Ultraschall als erste und einzige Maßnahme zur Darstellung eines Gelenkergusses genügen. Hingegen ist ein sog. **Hüftschnupfen**

**Figure 17:**
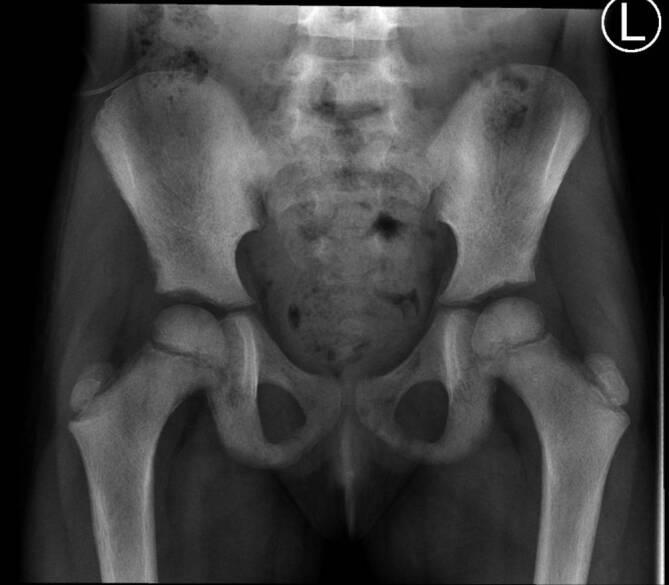

Hüftschnupfen

eine Ausschlussdiagnose, und man sollte immer an ein Frühstadium eines Morbus Perthes denken, an eine Epiphysiolyse, eine septische Arthritis oder einen Knocheninfarkt (z. B. Neuroblastommetastasierung oder Sichelzellanämie), sodass ein Röntgenbild häufig nötig sein kann (Abb. 17).

**Figure 18:**
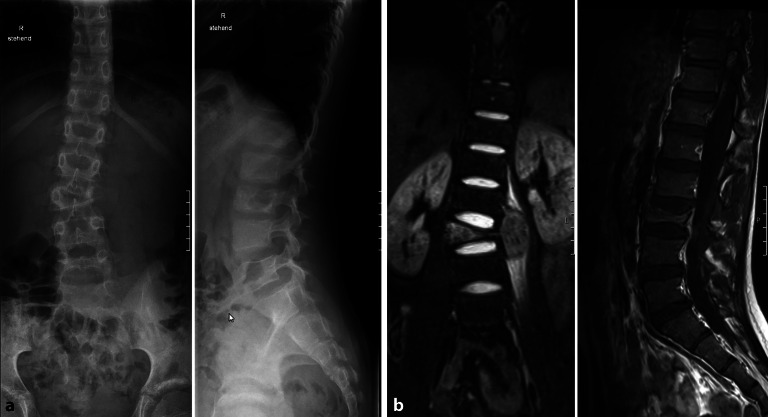

Die Indikation zu einer MRT bei Kindern mit **unklaren Rückenschmerzen**

unklaren Rückenschmerzen,

die im Röntgenbild nicht erklärt werden können, muss früh und großzügig gestellt werden z. B. zur Diagnosestellung Osteomyelitis, Spondylodiszitis oder Knochentumoren (Abb. 18).

Die MRT ist äußerst nützlich bei Kniebinnenläsionen, Schulterverletzungen oder Hüft-Impingement-Abklärungen. Arthrographische MR-Untersuchungen werden bei Kindern hingegen nur selten durchgeführt.

## Befundung des pädiatrischen Skeletts

Der radiologische Befund zu jeder Bildgebung soll eine akkurate und zeitnahe Interpretation der Bilder beinhalten und in einem Format erscheinen, das die formale Analyse oder Klarstellung der klinischen Fragestellung beschreibt. Der Text soll den Befund möglichst genau, klar und prägnant beschreiben, sodass eine **klinisch-pathologische Korrelation**

klinisch-pathologische Korrelation

möglich ist. Der Befund soll neben der Deskription eine Differenzialdiagnose und weitere Empfehlungen zur Abklärung enthalten, die für den Kliniker einen Behandlungsplan für den Patienten erlauben.

Die Befundung der bildgebenden Verfahren hat sich auch weiterentwickelt. Grundsätzlich soll für jede bildgebende Untersuchung die Indikation geprüft werden. Künftig können intelligente Systeme direkt aus der Bildinformation im PACS Messungen in das Radiologie-Informationssystem (RIS) integrieren. Der Befund muss weiterhin die Indikation, die klinische Fragestellung, den deskriptiven Befund und die Beurteilung enthalten. Dabei sollen die Art der Untersuchung und die Ebenen im Titel erwähnt werden.

Für die **Frakturbeschreibung**

Frakturbeschreibung

haben sich unterschiedliche Klassifikationen und Nomenklaturen etabliert. Grundlage gemäß der AO(Arbeitsgemeinschaft für Osteosynthesefragen)-Klassifikation nach Slongo et al. [10, 11] ist die anatomische Zuordnung nach Knochen und die Lokalisation innerhalb des Knochens in Epiphyse, Metaphyse und Diaphyse. Anschließend folgen die Art der Fraktur (Torus‑, Grünholz- oder komplette Frakturen) und schließlich das Ausmaß der Fehlstellung. Eine Sonderstellung des pädiatrischen Skeletts sind Frakturen, welche die offenen Epiphysenfugen betreffen (Salter-Harris- oder Aitken-Müller-Klassifikation), und bei beginnendem Verschluss der Wachstumsfugen des OSG müssen Übergangsfrakturen wie Twoplane- und Triplane-Frakturen unterschieden werden. Durch diese standardisierte Definition der Frakturen gelingt eine einfache Beurteilung in konservativ zu behandelnde Frakturen und Frakturen, die einer chirurgischen Reposition mit Fixation bedürfen [10, 11, 12].

Für die Beschreibung des Skeletts bei **Tumoren**

Tumoren

und **Entzündungen**

Entzündungen

spielt die Charakterisierung der ossären Läsion eine wichtige Rolle. Es wird bei **Osteolysen**

Osteolysen

geografisch in Mottenfraß- und permeative Osteolyse unterschieden. Die Periostreaktion kann Aufschluss geben über die Aggressivität einer Knochenveränderung (Codman-Dreieck, Spiculae, lamelläre Periostreaktion). Neben Osteolysen spielen **Osteosklerosen**

Osteosklerosen

eine Rolle bei chronischen Veränderungen oder Knochenmatrix-bildenden Tumoren. Die anatomische Lokalisation in Epiphyse, Metaphyse und Diaphyse, das Alter und Geschlecht können die Differenzialdiagnose von ossären Prozessen einschränken.

Viele neue **automatische Auswertesoftware**

automatische Auswertesoftware

-Lösungen erlauben eine standardisierte und untersucherunabhängige Vermessung des Skeletts. Mit künstlicher Intelligenz gelingt es, das Knochenalter der Hand verlässlich zu bestimmen. Längen‑, Winkel- und Kurvenmessungen können dank Segmentierung des Knochens in Orthoradiogrammen, Beckenröntgen oder Skolioseröntgen automatisch durchgeführt werden. Dennoch bedarf es der ärztlichen Kontrolle solcher automatischen Auswertungen, sodass Pathologien nicht übersehen werden.

Immer häufiger werden strukturierte Befundtexte und Befundvorlagen verwendet, die systematisch die Beschreibung aller relevanten Strukturen erlauben (z. B. Knie-MRT, Schulter-MRT, Rotationsmessungen etc.).

Bei allen dosisintensiven Untersuchungen wie Computertomographie, Durchleuchtungen und intraoperativen Bildverstärkeruntersuchungen müssen Aufklärungsgespräch, verwendete Dosis und Zeit sowie verwendete Kontrastmittelmenge und Name dokumentiert werden.

## Bildverteilung und Archivierung

Dank des „Picture Archiving and Communication System“ (**PACS**

PACS)

gehen Röntgenbilder nicht mehr verloren und sind innerhalb eines Klinikverbundes einfach und jederzeit abrufbar. Auch zuweisenden Hausärzten und Patienten können unmittelbar digitale Bildinformationen zugänglich gemacht werden. Als Bildformat hat sich der **DICOM**

DICOM

(„Digital Imaging and Communications in Medicine“)-Standard etabliert, der auch an einer beliebigen Workstation eine Nachbearbeitung wie Bildfensterung, Messungen und Rekonstruktionen erlaubt. Wegen der immer zahlreicheren Bilderdaten in der CT und MRT, aber auch im Ultraschall werden immer größere digitale Bildspeicher benötigt.

## Fazit für die Praxis

- Moderne digitale Röntgenanlagen ermöglichen eine deutlich niedrigere Strahlenbelastung bei besserer Bildqualität im Vergleich zu Computer Radiographie(CR)-Anlagen oder Film-Folien-Systemen. Gonadenschutze sind somit obsolet. Es gelten weiterhin bei allen bildgebenden Modalitäten mit ionisierenden Strahlen die Grundlagen des Strahlenschutzes im Kindesalter, wobei die Indikationsprüfung, das Einblenden, der Einsatz von Filtern und alters- bzw. gewichtsadaptierten Einstellparametern wesentlich zum Strahlenschutz beitragen können.
- Bei Kindern müssen die Eltern gut eingebunden und aufgeklärt werden. Positionierung der Gelenke, Kollimation und Optimierung der Dosisparameter spielen eine wichtige Rolle in der Kinderradiologie. Es soll immer daran gedacht werden, dass das Kind Schmerzen haben könnte und man deshalb unnötige Manipulationen vermeiden sollte, vielmehr sollte in Betracht gezogen werden, die Röntgenröhre zu schwenken.
- Ultraschall, Computertomographie oder Magnetresonanztomographie können ergänzend offene Fragestellungen beantworten, wobei jede dieser Modalitäten ihre Stärken und Schwächen hat. Das Röntgenbild bildet weiterhin die Grundlage der diagnostischen Bildgebung des pädiatrischen Skeletts.

## CME-Fragebogen

Bei Kleinkindern sollen bei Verdacht auf Unterarmfrakturen immer die beiden angrenzenden Gelenke geröntgt werden, um folgenden Befund besser beurteilen zu können:

Luxation des Radiusköpfchens bei isolierter Ulnafraktur

Subluxation des Radiusköpfchens bei distaler Radiusfraktur

Luxation im distalen Ulna-Radial-Gelenk

Bowing von Radius und Ulna

Luxation der Ulna bei proximaler Ulnafraktur

Ein indirektes Frakturzeichen bei Ellbogenfrakturen kann sein:

Das Pronator-Quadratus-Zeichen

Der anteriore Tiltwinkel der Tuberositas

Das Fat-Pad-Zeichen

Der dorsale Tiltwinkel der distalen Radiuswachstumsfuge

„Toddler sign“

Die Röntgendosis kann vermindert werden bei altersentsprechender Anpassung von:

Film-Folien Systemen

Dynamischen Durchleuchtungssequenzen statt Röntgenbild

einer Vergrößerung des Abstandes der Röntgenröhre

mAS (Milliamperesekunde) und kV (Kilovolt)

Dual-Energy-CT (Computertomographie) bei unklaren Fragestellungen

Die Sonographie ist dem Röntgenbild überlegen bei …

Dokumentation von Galeazzi Frakturen.

Sensitivität von intraartikulären Frakturen.

Winkelbestimmung von suprakondylären Frakturen.

Sensitivität von Gelenkergüssen.

Ausprägung der Achsabweichung bei Schaftfrakturen.

Die konventionelle Computertomographie von Gelenken ist hilfreich bei …

Gefäßrekonstruktion ohne Kontrastmittel.

Übergangsfrakturen bei Jugendlichen.

Darstellung von Knochenödemen.

Früherkennung einer Osteomyelitis.

Altersbestimmung von Wirbelkörperfrakturen.

Die Magnetresonanztomographie (MRT) wird bei Kindern zumeist *nicht* eingesetzt bei …

Torsionsmessungen der unteren Extremitäten.

Kniebinnenläsionen.

Beurteilung bei Hüftdysplasien nach Reposition im Gips (Typ IV).

Osteomyelitis der Metaphysen.

Frakturabklärung im Schockraum bei Polytrauma.

Eine der wichtigsten Strahlenschutzmaßnahmen beinhaltet bei Kindern:

Grundsätzlich beide Seiten zum Vergleich röntgen

Die Indikation zur Röntgendiagnostik prüfen

Alternativen wie Computertomographie (CT) oder Fluoroskopie anstatt Magnetresonanztomographie (MRT) oder Sonographie

Wenig Rauschen im Bild

Dezentrierung des Gelenkes in der Computertomographie (CT)

Welche Aussage trifft *nicht* zu?

Der Gonadenschutz bei Kindern wird nicht mehr empfohlen.

Der Gonadenschutz führt zu einer höheren Streustrahlung von Darm und Magen.

Der Gonadenschutz bei Mädchen schützt nicht immer die Ovarien im Becken.

Die Strahlenbelastung eines Röntgenbildes des Beckens entspricht ca. 100 Tagen natürlicher Strahlenexposition (1,2 mSv).

Die Strahlenbelastung des Röntgens des Fußes entspricht ca. 2 h natürlicher Strahlenexposition (0,001 mSv).

Welche Aussage ist richtig? Beim intraoperativen Gebrauch eines Bildverstärkers sollte man darauf achten, dass …

versucht wird, lange Durchleuchtungssequenzen anzustreben.

ein Einblenden des Operationsgebietes vermieden wird.

die maximale Vergrößerung gewählt wird.

das Lichtvisier vermieden wird.

eine kurze Pulsationsrate verwendet wird.

Welche Aussage trifft für Skolioseröntgenbilder zu?

Die Dosis kann mit Verwenden eines Kupferfilters um 10 % reduziert werden.

Die Optimierung von kV (Kilovolt) und mAs (Milliamperesekunde) ermöglicht zusätzlich eine Dosisreduktion von über 50 %.

Die Bildqualität ist erheblich beeinträchtigt bei einer Dosisreduktion von 50 % im Vergleich zu Erwachsenenparametern.

Die Kollimation der Brustdrüsen trägt über 20 % zur Dosisreduktion bei.

Die Wirbelsäule ist bei Kleinkindern weniger strahlensensibel als bei älteren Kindern.
